# Changing Epidemiology of Human Brucellosis, Germany, 1962–2005

**DOI:** 10.3201/eid1312.070527

**Published:** 2007-12

**Authors:** Sascha Al Dahouk, Heinrich Neubauer, Andreas Hensel, Irene Schöneberg, Karsten Nöckler, Katharina Alpers, Hiltrud Merzenich, Klaus Stark, Andreas Jansen

**Affiliations:** * Rheinisch-Westfälische Technische Hochschule Aachen University, Aachen, Germany; † Friedrich Loeffler Institute, Jena, Germany; ‡ Federal Institute for Risk Assessment, Berlin, Germany; § Robert Koch Institute, Berlin, Germany; ¶ Johannes Gutenberg University of Mainz, Mainz, Germany

**Keywords:** Human brucellosis, epidemiology, trends, Germany, Turkish immigrants, research

## Abstract

Trends in the epidemiology of human brucellosis in Germany were investigated by analyzing national surveillance data (1962–2005) complemented by a questionnaire-based survey (1995–2000). After a steady decrease in brucellosis incidence from 1962 to the 1980s, a persistent number of cases has been reported in recent years, with the highest incidence in Turkish immigrants (0.3/100,000 Turks vs. 0.01/100,000 in the German population; incidence rate ratio 29). Among cases with reported exposure risks, 59% were related to the consumption of unpasteurized cheese from brucellosis-endemic countries. The mean diagnostic delay was 2.5 months. Case fatality rates increased from 0.4% (1978–1981) to a maximum of 6.5% (1998–2001). The epidemiology of brucellosis in Germany has evolved from an endemic occupational disease among the German population into a travel-associated foodborne zoonosis, primarily affecting Turkish immigrants. Prolonged diagnostic delays and high case fatality call for targeted public health measures.

## Abstract 2

This endemic occupational disease has become a foodborne and travel-associated zoonosis primarily affecting Turkish immigrants.

Brucellosis is one of the most common zoonotic diseases worldwide (*1*). The disease is caused by *Brucella* spp. and is transmitted from its animal reservoirs to humans by direct contact with infected animals or, more often, through the consumption of raw animal products such as unpasteurized milk or cheese. Four of 6 nomen species of the genus *Brucella* are pathogenic for humans, i.e., *B. melitensis* (transmitted from sheep and goats), *B. abortus* (from cattle and other bovidae), *B. suis* (from pigs), and *B. canis* (from dogs) (*2*).

In Germany, human brucellosis was highly endemic from the 1950s well into the 1980s, with up to 500 cases reported annually. Most of these cases were related to occupational exposure associated with calf breeding and dairy farming, leading to a predominance of *B. abortus* infections (*3**,**4*). Because of successfully established eradication and control programs for animal brucellosis, the number of human cases decreased steadily. In 2000, Germany was declared “officially free from ovine/caprine and bovine brucellosis” (*5*). Human brucellosis cases, however, continued to occur in Germany. Although limited case series from Germany and Denmark indicate that human brucellosis could be associated with travel to and immigration from disease-endemic areas (*6**,**7*), there are no population-based or nationwide studies on epidemiologic characteristics of the disease in northern and central Europe.

The objective of our study was to describe trends in laboratory-confirmed human brucellosis in Germany over the past 40 years by analyzing national surveillance data. To provide background information, which may be useful for targeting public health measures, we focused on geographic origin and source of infection, modes of transmission, risk factors, and regional distribution of the disease.

## Methods

In the former German Democratic Republic (East Germany), human brucellosis became a reportable disease in 1951. From 1947 through 1961, in the former Federal Republic of Germany (West Germany) only *B. abortus* infections were reported. After 1962, brucellosis (independent of the disease-causing species) became a reportable disease according to the West German Federal Communicable Disease Act, which was the applicable law after the reunification in 1990.

Detailed data about brucellosis patients were compiled from 1995 through 2005 on demographics (age, sex, nationality, and current residence), onset of symptoms, clinical signs (fever, night sweats, fatigue, lack of appetite or weight loss, headache, arthralgia), contact dates with the treating physician, hospitalization, death, laboratory diagnosis, bacterial species, geographic origin, and possible vehicle of infection. The data collected from 1995 through 2000 are based on a standardized questionnaire, which was sent to local health departments for every reported case of brucellosis (*8*). Since 2001, similar information has been available from an improved surveillance system implemented for mandatory case reporting of infectious diseases. Fatal brucellosis cases documented on death certificates (1962–2005) were obtained from the Information System of Federal Health Monitoring, Germany (www.gbe-bund.de); population data were provided by the Federal Statistical Office, Germany (www.destatis.de).

Both clinical signs (the occurrence of an acute febrile illness or 2 other clinical signs) and laboratory confirmation (positive culture, only 1 significant titer, or an increase in the titer in the follow-up serum sample) were required to meet the case definition for brucellosis (*9*). From 1995–2005, isolates suspected to be *Brucella* spp. were sent from various microbiologic laboratories throughout Germany to the former German Reference Center for Human Brucellosis at the Federal Institute for Risk Assessment in Berlin. Standard microbiologic methods were used for further identification (*10*).

To assess temporal trends, mean annual incidences and case-fatality ratios were calculated for 4-year intervals starting from 1962 through 2005. Statistical tests for trend were performed by using the Cochrane-Armitage test (*11*); 95% confidence intervals were calculated according to Wilson (*12*). The Mann-Whitney test was used for comparative analysis of continuous variables. We tested for significance of incidence rate ratios (IRRs) using a Poisson regression model. Odds ratios (ORs) were tested for significance by using the χ^2^ test. Data were analyzed with EpiInfo version 6.04 (Centers for Disease Control and Prevention, Atlanta, GA, USA) and Stata version 9.0 (StatCorp., College Station, TX, USA). A p value <0.05 was considered significant.

## Results

**Figure 1:**
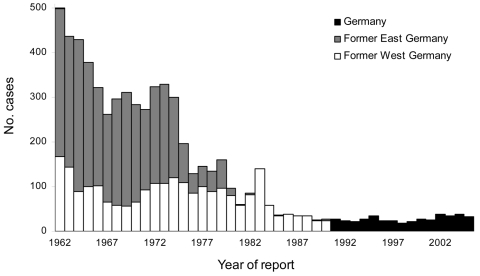
Reported brucellosis cases, Germany, 1962–2005.

**Figure 2:**
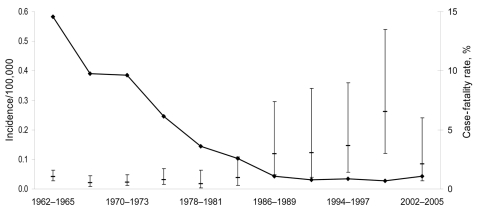
Incidence (per 100,000 inhabitants) and case-fatality rate for brucellosis, Germany, 1962–2005. Error bars indicate 95% confidence intervals.

From 1962–2005, 6,269 human brucellosis cases were reported in Germany. During this 44-year period, the annual number of cases generally declined (Figure 1). The mean annual incidence decreased from 0.6/100,000 population (1962–1965) to the lowest observed incidence of 0.03/100,000 population during 1998–2001 (Figure 2). A total of 58 deaths were caused by brucellosis (overall case-fatality rate 0.9%). The lowest case-fatality rate was 0.4% in 1978–1981. From then on, a significantly increasing trend (p<0.01) reaching a maximum of 6.5% in 1998–2001 was observed, which subsequently dropped to 2.1% in 2002–2005.

**Figure 3:**
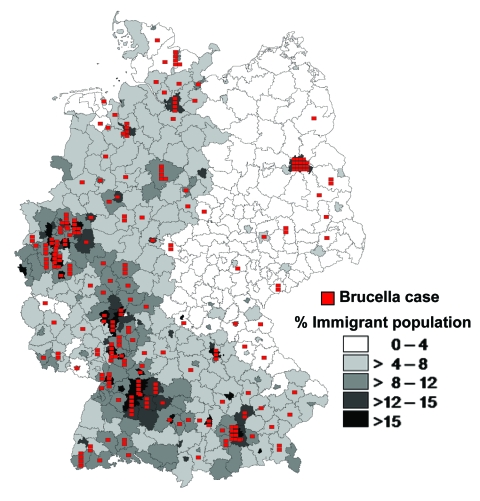
Regional distribution of brucellosis cases and percentage of immigrants per county, Germany, 1995–2005.

Through 1974, most of the brucellosis cases were reported from East Germany, with a maximum of 82% in 1969. After 1974, the relative number of cases reported in East Germany decreased compared with those in West Germany. Since 1981, brucellosis has been rarely reported in East Germany; the West-East divide was still present after reunification (Figure 3).

From 1995 to 2005, a total of 290 brucellosis cases were reported, of which 245 (84%) met the case definition and were included in this analysis. Area of residence, sex, age, clinical symptoms, and laboratory findings were known for all 245 case-patients. Most cases were reported in the federal states of North Rhine Westphalia (49), Baden-Württemberg (45), Bavaria (39), and Hesse (23); in the cities of Berlin (19), Hamburg (8) and Bremen (4); and in large conurbations, e.g. Munich (10) and Ludwigshafen (8). The spatial distribution of brucellosis cases was associated with the immigrant density in the administrative districts (Figure 3).

**Figure 4:**
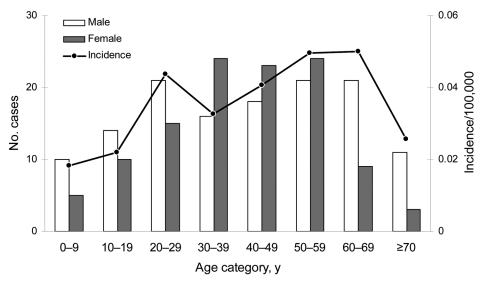
Age and sex distribution of brucellosis cases (n = 245), Germany, 1995–2005.

Both sexes were almost equally represented among brucellosis patients (54% male vs. 46% female). In patients <30 years of age and >59 years of age, male sex predominated (60% and 73%, respectively); in persons 30–59 years of age, 56% were female. The age-specific incidence was highest for persons 60–69 years of age, with a mean annual incidence of 0.05/100,000, and lowest for children <10 years of age, with a mean annual incidence of 0.02/100,000 (Figure 4).

Detailed data about the nationality of patients were available for 106 (43%) of the 245 cases. A total of 58 (55%) were non-Germans, of which 62% (36) were Turkish. Four were Italian, 3 each were Greek and Iraqi, 2 were Kazakh, and 1 each were Bosnian, Kosovar, Portuguese, Syrian, Arabian, Indian, Pakistani, Yemeni, and Somali; in 1 case, a non-German status was reported without nationality. The incidence rate was 0.3/100,000 in Turks and 0.01/100,000 in Germans (IRR 29, p<0.01).

The country where the infection had been contracted was known for 234 (96%) of 245 cases. In 172 cases (74%), the origin of infection was likely to be outside Germany. Of these, 137 (80%) were associated with travel to disease-endemic countries surrounding the Mediterranean Sea, e.g. Turkey (94), Italy (13), and Spain (9). Possible origins of infection were the Balkans for 5 cases, African countries for 7 cases, Middle Eastern countries (not bordering the Mediterranean Sea) for 6 cases, Minor Asian countries for 9 cases, former Soviet Union countries for 5 cases, and the Czech Republic for 1 case. Two patients were infected overseas (Peru and New Zealand). In 62 cases (26%), the origin of infection was assumed to be Germany.

In 102 (42%) of 245 patients, >1 probable source of infection could be identified. Fifty-six (55%) had only 1 exposure risk, whereas the other 46 patients (45%) mentioned various combinations. Twenty-seven patients consumed unpasteurized milk, 65 patients ate unpasteurized cheese or other dairy products, and 7 patients ate raw meat. Foodborne infections were almost equally distributed among Turks and Germans (31% vs. 35%). Direct contact with cattle, sheep and goats was reported by 16, 24, and 16 patients, respectively. Most of the people infected by direct animal contact were Turks (49%); only 29% were Germans.

In 18 cases (18%), a possible occupational exposure was reported. Among these, 7 infections were laboratory-acquired, exclusively in German cases. The other work-related cases were linked to direct contact with animals or animal products outside Germany. Two shepherds, 2 persons working on a sheep breeding farm, 2 farmers, 4 butchers and 1 veterinarian were affected. In 84 cases (82%), no occupational exposure risk was observed.

Ten minor outbreaks were reported during 1995–2005. Four cases were epidemiologically linked to *Brucella* infections observed in friends and relatives living in disease-endemic countries, i.e., Turkey, Italy, and Bosnia. In 7 cases, the patient was related to at least 1 other person living in Germany who also had *Brucella* infection. One laboratory-acquired infection and its index case were also reported as an outbreak.

**Figure 5:**
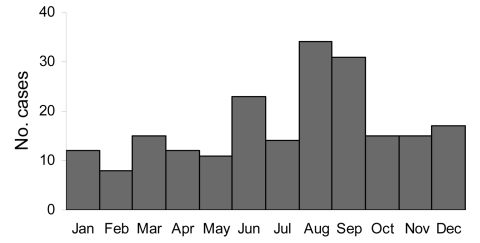
Seasonal distribution of brucellosis cases (n = 207), Germany, 1995–2005.

The date of onset for symptomatic disease was reported for 207 (84%) of 245 cases. In most cases, the onset of disease was in August or September (31%). Another smaller peak comprising 23 cases (11%) occurred in June (Figure 5). In 85 cases, more detailed information about the incubation period was available. The period between presumed infection and onset of symptomatic disease varied extremely, ranging from a few days to 24 months (median 4 weeks).

The major symptom in 215 (88%) of 245 patients was fever, which was significantly associated with hospitalization of the patient (OR 4.1; p<0.01). A total of 121 (49%) patients reported fatigue; 105 (43%) reported arthralgia, 101 (41%) reported headaches, 66 (27%) reported massive sweating, 30 (12%) reported loss of appetite, and 31 (13%) reported loss of weight.

The delay between onset of disease and definite laboratory-confirmed diagnosis was reported for 175 cases. The mean diagnostic delay was 2.5 months, with no differences between ethnic groups. In 77 patients (44%), brucellosis was diagnosed within 4 weeks. For 89 patients (51%), diagnostic delay ranged from 1 to 6 months. In the remaining 9 patients (5%), brucellosis was definitely diagnosed >6 months after onset of symptoms.

In 24 cases, the period between the first presumptive diagnosis and the final laboratory confirmation was reported; the mean period was 6 days. Sixty-three (26%) patients were treated as outpatients, while 181 (74%) were hospitalized. In 1 case, this information was not available.

From 1995–2005, a total of 134 cases was culture-proven at the former German Reference Centre for Human Brucellosis at the Federal Institute for Risk Assessment in Berlin. Standard microbiologic tests identified 131 *B. melitensis* isolates (98%), 1 *B. suis* strain, and 2 *B. abortus* strains. Of the 245 total cases, 164 (67%) were diagnosed by serologic tests, 113 (69%) by serum agglutination test (SAT), 3 (2%) by complement fixation (CFT), and 15 (9%) by ELISA. In 31 cases (19%), a positive SAT was confirmed by CFT or ELISA. In 2 cases, the serologic tests used could not be identified. Among the cases with serologic confirmation (n = 164), 1 strongly elevated titer was reported for 81 patients, while seroconversion was shown in 11 patients. In 72 cases, no data on the type of serologic confirmation test was available.

## Discussion

Up to the mid-1980s, a substantial decrease in the incidence of human brucellosis was observed in Germany. However, national surveillance data demonstrated a persistent level of reported cases in recent years. Our study indicates that these infections are primarily related to persons with a migrational background. Taking into account that <10% of *Brucella* infections are recognized and reported because of unspecific clinical symptoms (*13**,**14*), our results strongly suggest that human brucellosis has emerged as an important and probably neglected health problem among immigrants in Germany. The present epidemiology of brucellosis in Germany mirrors the reemergence of the disease in Turkey. An increase in brucellosis incidence has also been reported from several other countries in the Middle East and the Balkans (*1*), which emphasizes the magnitude of the problem and its potential to accelerate in the future. Immigrants from these regions form an increasing proportion of the German population.

The current status of brucellosis in Germany is the result of continuous changes in the epidemiologic characteristics of the disease during the past 40 years. The number of reported autochthonous human cases continuously decreased in parallel to the decreasing prevalence of infected animals. At the same time, the number of immigrants, especially from Turkey, increased considerably. In 1960, only 1% of the German population was foreign born and only 2,700 Turkish residents lived in Germany. In 2004, ≈8.8% of the population was foreign born, and Turks formed the largest foreign nationality group, with 1,764,318 immigrants (24% of all foreigners).

Our results indicate that the exposure risk of Turkish immigrants to *Brucella* spp. continues after immigration to Germany, with a brucellosis incidence (0.3 cases/100,000 Turkish immigrants) falling between the incidence in the German population as a whole (0.01 cases/100,000 Germans) and the incidence observed in Turkey (26.2 cases/100,000 population) (*1*). The continuing risk may be attributed to more frequent exposures during summer recreational activities in disease-endemic countries, e.g., when visiting friends and relatives in rural areas. In brucellosis-endemic regions, the peak for human brucellosis is in June and July (*15**–**17*). Onset of disease occurs in August and September, just after the end of the German summer holiday season, in most reported cases. In addition, *Brucella* spp. may survive for several days up to months in contaminated food products privately imported from disease-endemic countries (*18*), which may contribute to infections contracted in Germany. An association of brucellosis with the immigrant population has previously been reported from Denmark and the United States (*7**,**19*). In the United States, Hispanic ethnicity, recent travel to disease-endemic areas in Mexico, and ingestion of nonpasteurized dairy products are major risk factors for *Brucella* infections (*13**,**19**–**22*).

Brucellosis was traditionally more prevalent in German states with a high degree of agricultural activity. Our results demonstrate a fundamental shift of brucellosis from a rural disease into an infection of urban and suburban residents. Because most immigrants live in the centers of industry, most cases were reported from cities and areas with high-density populations in Germany. The pronounced West-East divide we observed mirrors the much higher proportion of foreign-born people in western Germany compared with eastern Germany (10.1% versus 2.4%).

Regarding the age distribution in our study group, only 16% of the reported cases were <20 years of age. The age-specific incidence was highest in persons 60–69 years of age. These persons were mainly first-generation immigrants who keep in closer contact with family members still living in their homelands. A similar age-specific incidence distribution has been described in studies from other countries not endemic for brucellosis, whereas in brucellosis-endemic countries, cases of this disease do not cluster in a particular age class (*16*). We did not observe a male predominance in the working age group as in countries where brucellosis is strongly related to occupational exposure risks. In Germany, brucellosis has evolved into a foodborne disease, and unpasteurized goat cheese is the most frequently reported vehicle of exposure in our study population; thus, there is no reason to expect gender predominance. From 1995–2005, 2.9% of the cases reported were associated with *Brucella* infections in family members. The serologic screening of household members of brucellosis patients may therefore help to detect these frequently unrecognized cases with identical risk factors (*23*).

In Germany, ≈7% of the infections with a known source were laboratory acquired. *Brucella* spp. are among the most commonly recognized causes of laboratory-transmitted infections worldwide, but only 2% of all human cases are actually laboratory-acquired (*13**,**24*). This discrepancy may reflect that microbiologists in German laboratories are not aware of brucellosis as a possible case of fever of unknown origin because the disease is very rare in Germany. A low index of suspicion and misidentification of the organism may lead to a higher proportion of laboratory-associated infections.

Consistent with the literature, fever >38.5°C was the leading symptom in most (88%) of our patients. Osteoarticular manifestations are known to be the most frequent focal complications (*17**,**25**,**26*) and were reported in 43% of our cases. Key results of our study are the extensive diagnostic delay in brucellosis and the exceptionally high case-fatality rate. The degree of illness in patients with fever of unknown origin is directly related to the diagnostic delay. In 56% of the cases reported in Germany, symptoms lasted >4 weeks before diagnosis, and the mean diagnostic delay was 2.5 months. It is well documented that the number of focal complications increases with a diagnostic delay of >30 days and the risk for an unfavorable clinical course is much higher in patients with focal complications (*25*). In disease-endemic areas, the index of suspicion is high, and the duration of symptomatic disease before hospital admission is <2 weeks in 72% of the cases (*27*). In part, the increase in deaths observed in our study may be caused by a lack of suspicion by medical professionals. In addition, language barriers may hinder obtaining detailed medical histories from immigrants (*28*).

Most human brucellosis cases worldwide are caused by *B. melitensis* (*29*), which is also true in Germany (98% of all isolates). Most *B. melitensis* strains isolated in Germany are of the East-Mediterranean genotype (*30*), which is consistent with the epidemiologic data presented.

## Conclusions

In Germany, brucellosis has emerged as a disease among Turkish immigrants. In this population group, the infection is associated with major diagnostic delays, possibly resulting in treatment failures, relapses, chronic courses, focal complications, and a high case-fatality rate. Because of a lack of knowledge on the changing epidemiology of the disease, many physicians may not be able to act efficiently as first responders recognizing natural or artificial outbreaks. Public health programs should therefore focus on educating the Turkish segment of the German population about the risks of consuming animal products imported from Turkey or unpasteurized cheese and other dairy products during visits to Turkey. In addition, healthcare providers should be informed about the disease, especially if they work in areas with a large Turkish population.
